# Radiation-induced leiomyosarcoma of the oropharynx

**DOI:** 10.1186/1746-1596-1-22

**Published:** 2006-08-22

**Authors:** Jens Pfeiffer, Carsten Christof Boedeker, Gerd Jürgen Ridder, Wolfgang Maier, Gian Kayser

**Affiliations:** 1 Department of Otorhinolaryngology – Head and Neck Surgery, Freiburg Medical School, Freiburg, Germany; 2 Institute of Pathology, Freiburg Medical School, Freiburg, Germany

## Abstract

Leiomyosarcoma is a malignant mesenchymal tumor originating from smooth muscle cells, which most frequently develops in the myometrium and in the gastro-intestinal tract. Reviewing the international literature, radiation-induced sarcoma arise in 0.035 to 0.2 % of all irradiated patients. Especially in the head and neck region, radiation-induced leiomyosarcoma is an extremely rare lesion. The authors report a case of a radiation-induced leiomyosarcoma of the tonsillar region of the oropharynx in a 51-year-old male patient, who had undergone radiation therapy of this region 38 years before. The lesion was treated by radical surgery. Diagnostic steps, histological presentation and therapy are described in detail and the literature concerning radiation induced malignancies in general as well as radiation induced leiomyosarcoma in particular is reviewed. The highlights of this case are an extremely uncommon location and a rare pathological entity of radiation induced malignancies.

## Background

Leiomyosarcoma is a malignant mesenchymal tumor which originates from smooth muscle cells and accounts for about 7 to 9 % of all soft tissue sarcomas [1-3]. Leiomyosarcomas most often arise in middle-aged persons, but it is extremely rare in adolescents and children [1]. Sites of involvement are primarily the myometrium and the gastro-intestinal tract, followed by the soft tissue of the extremities and the retroperitoneum [1]. Leiomyosarcoma of the head and neck is rare, as smooth muscle is seldom encountered in this region, mainly in the walls of blood vessels and the erector pili musculature of the skin [8-10].

Radiation-associated sarcomas are uncommon, constituting less than 5 % of all sarcomas, and generally associated with a poor prognosis [4]. Reviewing the international literature, radiation-induced sarcoma arise in 0.035 to 0.2 % of all irradiated patients [4]. The most common histologic subtypes of radiation-induced sarcomas are osteogenic, malignant fibrous histiocytoma, angio- and lymphangiosarcoma as well as spindle cell sarcoma [4,5,7]. Radiation-induced leiomyosarcoma is an uncommon histological entity. Especially in the head and neck region, this radiation-associated malignant neoplasm represents an exceptional rarity [5-8].

## Case presentation

**Figure 1:**
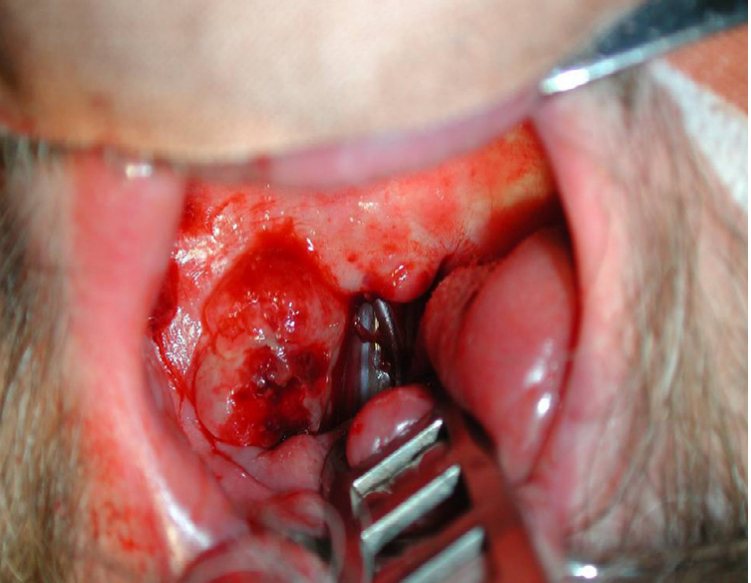
**Intraoperative view of the oropharynx**. A smooth bounded nodular tumor appears in the right-sided tonsil's region of the oropharynx. The neoformation expands on the anterior faucial pillar of the soft palate and the glosso-tonsillar furrow.

**Figure 2:**
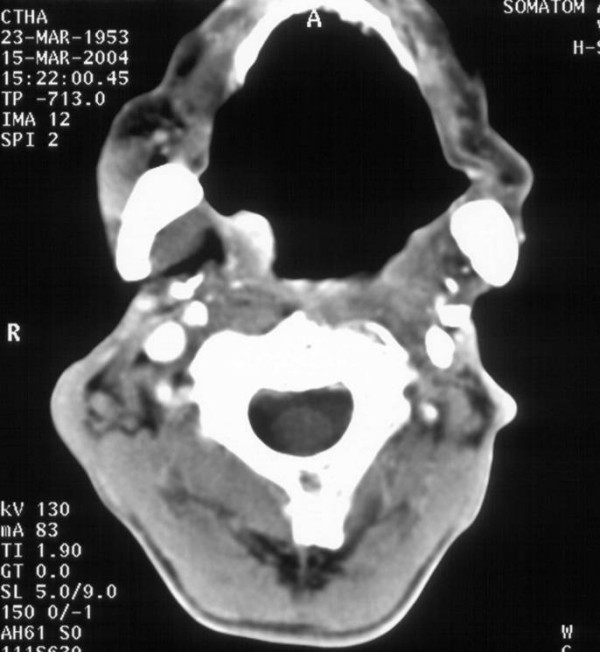
**Computed tomography (CT) of the oropharynx with contrast medium**. The axial projection shows a smooth bounded nodular neoformation in the right-sided tonsil's region of the oropharynx. The other layers of the tomography didn't reveal signs of enlarged cervical lymph nodes.

In March 2004 a 51-year-old male patient was referred to our outpatient's department because of oropharyngeal pain of the right tonsillar region. The complaints had started one year before with a right sided feeling of pressure in the oral cavity and the neck. In 1966, 38 years before the patient presented to our department, he had been treated for a squamous cell carcinoma of the left tonsil in the territory of the former Soviet Union. At that time, the then 13-year-old boy underwent radical surgery followed by adjuvant radiotherapy with a cumulative radiation dose of 101 Gray. Although the patient was only 13 years old at the time when the diagnosis of a squamous cell carcinoma of the left tonsil was made, the presented documents left no doubt about the initial histological diagnosis. When the 51-year-old patient presented to our department in 2004, the otorhinolaryngological examination showed a smooth bounded neoformation in the region of the right-sided tonsillar fossa and soft palate with a diameter of about 2.5 cm (Fig. 1). The tumor's palpation was dense. Computed tomography (CT) of the oropharynx confirmed the neoformation to be smooth bounded and well separated from the surrounding tissue (Fig. 2). Computed tomography and ultrasonic examination of the neck did not reveal enlarged cervical lymph nodes that might have indicated lymphogenic metastatic spread. No indication for hematogenous metastatic spread was found on plain x-rays of the chest, ultrasonographic examination of the abdomen and positron emission tomography imaging studies.

**Figure 3:**
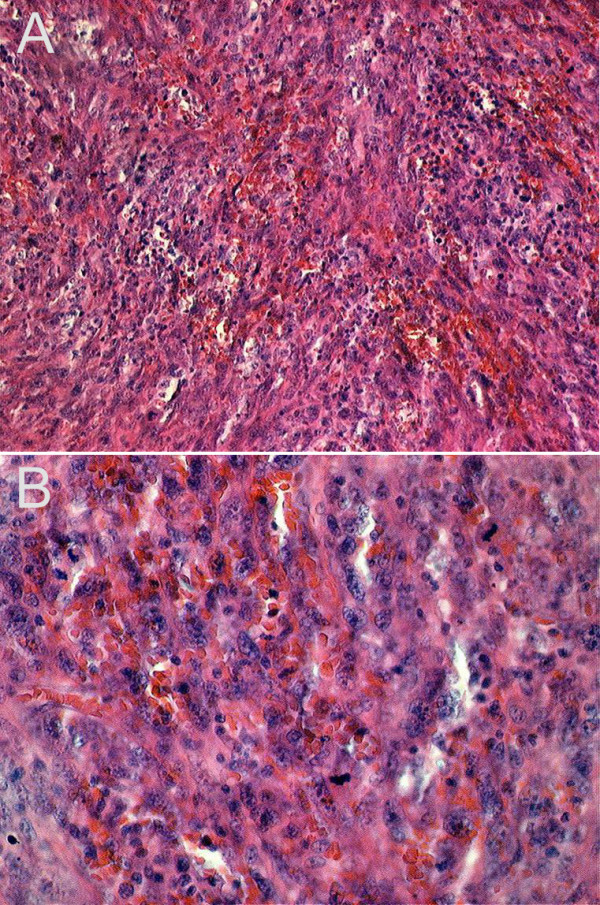
**A biopsy of the tumor in Hematoxilin-Eosin (HE) staining**. Spindle cell tumor with cellular polymorphism, mimicking a storiform growth pattern. Mitoses are also frequent supporting a malignant proliferation. HE 100 × (a), 200 × (b).

**Figure 4:**
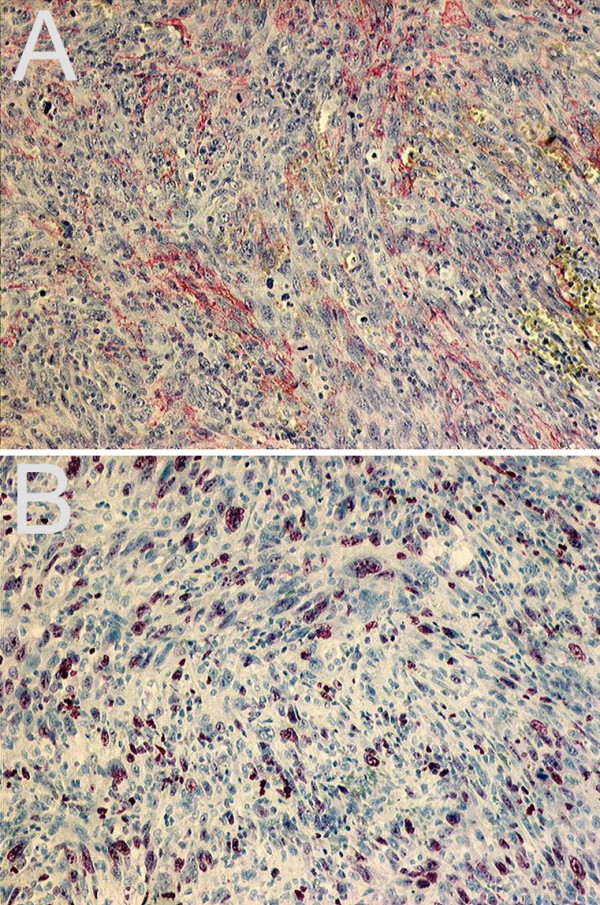
**Immunohistochemistry of a tumor-biopsy**. Immunohistochemical detection of a-actin, supporting the myogenic origin of the tumor (a, α-actin, 100×). The high proliferative activity is verified by MIB-1 (b, MIB-1, 100×).

An endoscopic examination of the larynx and hypopharynx was performed in general anesthesia and revealed these regions to be without pathological findings. Tumor-resection was then carried out by a transoral approach in combination with a mapping of the oropharyngeal mucous membrane. Histological examination of the tumor revealed spindle cells with marked polymorphism implicating a storiform growthpattern (Fig. 3 a,b). Via immunohistochemistry focal expression of α-actin and S100 could be detected (Fig. 4a). The proliferation-index was high (70 %) (Fig. 4b). Upon these morphologic and immunohistochemical findings a leiomosacroma of the oropharynx was diagnosed. The tumor was completely removed by surgical resection and histological examination revealed the resection margins to be free of tumor cells. Two months postoperatively an endoscopic control-investigation with biopsy collection was carried out for evaluation of microscopic tumor relapse. Histological examination revealed marginal tumor-extension in the region of the left-sided soft palate. Re-operation was carried out by laser-surgery of the left-sided soft palate and histological examination revealed no tumor-remnants to be left in situ. The course of disease and treatment was then supervised by clinical and radiological follow-up control-investigations at regular 3 months intervals. Unfortunately, a biopsy taken from the middle of the soft palate in June 2005 again revealed microscopic signs of tumor-relapse. At that time the patient was free of symptoms and did not show a clinical correlate of tumor-recurrence. Surgical resection of these findings had to be performed two more times and histological investigations finally revealed an R0-resection status. Up to now, the patient did not undergo chemotherapy or radiotherapy of the radiogenic-induced leiomyosarcoma. Close clinical follow-up control-examinations with biopsies of the former tumor region will now be performed. In case of new tumor-relapse radical surgical resection and defect reconstruction by a free radial forearm flap will be the treatment of choice.

## Discussion

Radiation therapy is a well accepted modality of treatment in the therapeutical scheme of head and neck cancer. It is of significance as an adjuvant method of treatment after surgery and in some cases a valuable alternative to surgery in cases where tumor-resection can not be performed or would lead to limited quality of life from mutilation and loss of function. Radiation-induced malignancy is a long-term complication of radiation therapy. The potential of ionizing radiation to induce malignant neoformations was already observed in the early 20^th ^century, only a short time after Roentgen first discovered x-rays. The first cases of radiation-induced skin cancers were reported by Frieben et al. in 1908 [11]. Criteria for diagnosing malignancy as radiation induced were firmly established by Cahan et al. in 1948 and first described for radiation-induced sarcomas of long bones [12]. These criteria comprised, that 1.) the patient had undergone radiation therapy, that 2.) the radiation-induced malignancy arose in the previously irradiated field, that 3.) there is histological evidence of a sarcoma, that 4.) there is a latency period of at least 5 years between radiation and the presentation of the radiation-induced sarcoma and, to exclude tumor relapse, that 5.) the proof, that primary and secondary tumor are of a different histological entity [4,6-8,12]. For almost 50 years these criteria formulated by Cahan et al. were the guiding principles for radiation-induced malignancies. Nevertheless, several small studies and case reports in the international medical literature described radiation induced malignancies originating from almost all kinds of tissue with a latency period between 3 and 45 years [6,7]. Furthermore, orthovoltage radiation, largely used prior to 1960, resulted in a much higher absorbed dose in bone than in soft tissue and explained, that bone sarcomas occurred with greater frequency than did soft-tissue sarcomas [4]. With the technological advance in radiation therapy, this relation changed and thus, Cahans' criteria were revised by Murray et al. in 1999 concerning the limitations on the latency period and the histological entity of radiation-induced neoformations [4,13]. Murray et al. included soft tissue sarcomas as well as radiation-associated malignancies, that arose after a latency period of less than five years, to fulfill the criteria for being radiation-induced [6,13]. Although the median latency period in studies reported in the international literature is 10 to 15 years, Brady et al. reported as much as 15 % of their patients developed sarcomas less than 5 years after radiation [4]. As the latency period appears to be inversely related to age, especially a significant percentage of older patients tend to have a latency period of less than 5 years [4,13]. Data suggest, that there is no relationship between cumulative radiation dose and the interval between exposure to radiotherapy and the development of radiation-induced sarcoma [5]. Surveying the international literature there are today no definitive criteria concerning the histological tissue of origin for radiation-induced malignant neoformations, but most data are still generated from sarcomas [6]. Several mechanisms in which radiation may induce genetic changes leading to malignant transformation are under discussion, but the exact development of radiation induced malignancy is still unclear. The loss of heterozygosity in directly radiated cell nuclei activate mutational occurrences in tumor suppressor genes resulting in malignant degeneration. Furthermore, genetic mutations may also be induced by cytoplasmatic irradiation and the release of cytokines. Therefore, important genetic effects can also be observed in cells, that did not directly receive nuclear radiation [6,14].

With an estimated incidence of 0.4 to 1.0 % radiation induced malignancy is a rare entitiy [6]. Radiation-associated sarcomas in particular are uncommon, constituting less than 5 % of all sarcomas [4,7]. Brady et al. reported, that the incidence of sarcomas that were associated with radiation ranged from 1.5 to 3.9 % for any investigated year [4]. According to his data, estimates of the incidence of sarcoma occurring in patients who received radiotherapy would suggest that radiation-induced sarcomas occur in 0.035 to 0.2 % of patients who receive radiation therapy [4]. These data are consistent with the information given by Demirkan et al.: In his report the risk of developing radiation-induced soft tissue sarcoma for a cancer patient who had to undergo radiotherapy and survives for more than 5 years is 0.1 %. Patel et al. also numeralizes the cumulative incidence of sarcoma after radiation therapy ranges from 0.03 to 0.3 % [5,8,15,16]. Despite this low incidence of radiation-induced sarcoma, this pathological entity is today expected to be seen more frequently, due to an increased life expectancy with progressive aging of the population combined with improved survival in cancer patients as a result of increased effectiveness of cancer therapy and better treatment regimes [5,8]. Neither a minimum of cumulative radiation dose nor a correlation between modality and form of radiation and the incidence of radiation-induced sarcomas is reported in the international medical literature [7]. A radiation dose-response relationship was demonstrated for all sarcomas and, for the first time in humans, for soft tissue sarcomas in the studies by Wong et al. in 1997 [5,8,17].

Within the head and neck region, practically all types of cancer have been reported after radiation therapy, but the skin and the thyroid are the most commonly involved tissues [5]. Squamous cell carcinoma is the most commonly reported histological entity, but it has to be emphasized that it is difficult to implicate therapeutic irradiation in the causation of head and neck squamous cell carcinomas because of the inherent risk of multiple primary tumors and the role of tobacco and alcohol in the development of head and neck carcinoma [5,18]. Although radiation-induced sarcoma is a well-documented long-term complication of radiation therapy for other sites, the head and neck is less commonly affected. Less than 1 % of all radiation-induced sarcomas arise within the head and neck region and thus is a rare entity with few reported series in the international literature [4,5,7]. Nevertheless the regular appearance of case reports in medical literature reflects the interest in these tumors as a long-term complication of radiation therapy in the head and neck region. The given details concerning the percentage of histological subtypes of radiation induced sarcomas vary slightly between the reported studies on topic. According to these reports the most common histologic types of radiation-induced sarcomas are considered to be osteogenic, malignant fibrous histiocytoma, angio- and lymphangiosarcoma as well as spindle cell sarcoma [4,5,7,8,19].

Leiomyosarcoma is a malignant spindle-cell mesenchymal tumor originating from smooth muscle cells and constituting about 7 to 9 % of all soft tissue sarcomas [1-3,10,20]. Leiomyosarcoma most often arises in middle-aged persons, but is extremely rare in adolescents and children [1]. It most frequently develops in the myometrium and in the gastro-intestinal tract, followed by the soft tissue of the extremities and the retroperitoneum [1-3]. Leiomyosarcoma of the head and neck is rare, as smooth muscle is seldom encountered in this region, mainly in the walls of blood vessels and the erector pili musculature of the skin [9,10,21-23]. Dry et al. in 1999 investigated leiomyosarcomas of the oral cavity and stated fewer than 50 cases reported in the English literature at the time of writing [9]. Leiomyosarcoma is not a lesion with a marked propensity for dissemination and metastatic spread. If it occurs, leiomyosarcoma like sarcoma in general typically metastasizes via hematogenous routes to sites such as lung, liver, bone and soft tissue [9,10,23]. According to Dry et al. hematogenous spread can be found in 34 % of the cases at the time of diagnosis [9]. Lymph node involvement is reported to be extremely rare. Interestingly, data presented by Dry et al. suggest, that leiomyosarcoma of the oral cavity, unlike leiomyosarcomas in soft tissue elsewhere, may occasionally metastasize to regional lymphnodes [9]. Particularly because of their rarity, leiomyosarcoma of the head and neck region may easily be mistaken for other more common spindle cell lesions in this location. Histologically, especially the tumors of the neural sheath and malignant melanoma of spindle cell type as well as spindle cell squamous carcinoma and spindle cell myoepithelioma must be included in the differential diagnosis and may mimic leiomyosarcoma in hematoxylin and eosin stained sections [9,10]. Also clinically benign lesions that may occur in the head and neck region like infantile and adult myofibroma and angioleiomyomas must be distinguished from leiomyosarcomas as they histologically may present similar features [9]. Immunohistological examinations and careful histological analysis are helpful adjuncts to distinguish between these entities. By immunohistochemistry leiomyosarcoma is positive for smooth muscle-specific α-actin [8-10]. Diagnosis of leiomyosarcoma is established in combination with the histological growth pattern of these spindle cell tumors which are storiform alignment of the spindle cells and marked polymorphism. A high proliferative activity is not uncommon.

In contrast to sarcomas of other histological subtypes, leiomyosarcomas are very rarely induced by radiotherapy [4,5,7]. Especially in the head and neck region, this radiation-associated histological entitiy is exceptionally uncommon. Demirkan et al. in 2003 found 23 cases of radiation-associated leiomyosarcomas reported in the international literature [7]. Clinical diagnosis of radiation-induced leiomyosarcoma can be difficult due to induration and fibrosis of the tissue within the former field of radiation [5]. Symptoms of leiomyosarcoma arising in the head and neck region are usually nonspecific. The most significant symptoms of radiation-induced sarcomas is the mass as well as the appearance of or the change in the character of pain in the irradiated area [5]. According to most reported studies on radiation-induced sarcomas complete surgical excision offers the only realistic chance for long-term survival and also appears to present the best means for palliation [4-8]. The response to radiotherapy of leiomyosarcoma in general is poor, especially when previous history of irradiation is taken into account for radiation-induced leiomyosarcomas [7,10,23]. Experience with adjuvant chemotherapy with Ifosfamide, Doxorubicin, Cisplatin, Adriamycin and Vinblastin in the treatment of radiation-induced sarcoma is limited but disappointing with the exception of the group of young patients with osteosarcoma, who seem to respond favourably to the treatment with methotrexat [4,6]. As surgical excision of radiation-induced sarcoma of the head and neck remains the only definitive treatment option, early diagnosis and a correspondingly better chance of complete surgical resection is essential for the patient's prognosis. Widely variable latency periods of radiation-induced sarcomas therefore necessitate lifelong monitoring of the radiated field, especially in young patients [6]. The prognosis of leiomyosarcoma arising within the head and neck region is poor, even if they are not associated with radiotherapy. It is reported that the survival rates are better in the gastrointestinal tract and the skin [10,23]. Radiation-induced sarcomas in particular are generally associated with a poor prognosis. Data suggest, that the overall five-year survival rate of radiation-induced sarcoma ranges between 10 and 30 % [4-6,19]. Despite these tumors having traditionally been associated with a poor prognosis, Brady et al. state, that the prognosis of patients with soft-tissue radiation-induced sarcoma of the extremities is similar to those of patients with spontaneous soft-tissue sarcoma of the extremities and that radiation-induced sarcoma in general have similar prognostic determinants to spontaneous sarcomas [4]. Thus, there is no evidence, based on the data presented by Brady et al. that patients with radiation-induced sarcomas should be excluded from clinical trials based on a worse prognosis [4]. The overall outlook concerning radiation-induced sarcomas of the head and neck in particular has also been reported to be very bleak [5-7]. The prognosis of radiation-induced sarcoma of the head and neck region is generally perceived to be worse than spontaneous sarcomas of the head and neck of similar stage and also appears to be worse than the prognosis of radiation-induced soft-tissue sarcoma and osteogenic sarcoma of similar stage in other sites [5]. Although van der Laan et al. state that survival data after treatment of radiation-induced tumors is not different from nonradiation-induced tumors in the head and neck, most papers quote the poor outcome of radiation-induced sarcoma of the head and neck with reported five-year disease free survivals of less than 10 % [5,24]. According to Patel et al. the poor prognosis of radiation-induced sarcoma of the head and neck may be explained on the basis of a delay in diagnosis due to a difficult clinical examination and nonspecific symptoms in an irradiated tissue, the limits of surgical resection within the head and neck region as well as limited treatment options and a relatively poor sensitivity of these tumors to chemotherapy [5].

We present here an additional case of radiation-induced leiomyosarcoma of the head and neck. The presented case fulfils all criteria for diagnosing the malignancy as radiation induced, that were formulated by Cahan et al. in 1948 and that are described above [12]. The neoplasm occurred as a long-term complication of cancer treatment with a latency period of 38 years after radiation-therapy with an excessive cumulative radiation dose of 101 Gray in a young patient. Although squamous cell carcinoma of the oropharynx is a rare lesion at the age of 13, the presented documents left no doubt about this histological diagnosis of the primary tumor and tumor relapse of a leiomyosarcoma 38 years after treatment appears hardly possible. Furthermore leiomyosarcoma is an extremely uncommon neoplasm in adolescents and children [1]. While the primary squamous cell carcinoma arose in the left tonsil, the presented radiation-induced sarcoma became apparent in the right-sided oropharynx and was successfully treated by radical surgery until now.

## Conclusion

The presented case demonstrates, that clinicians managing cancer patients as well as pathologists have to be aware of radiation-induced sarcomas as a long-term complication of treatment, especially as this pathological entity is today expected to be seen more frequently due to an increased life expectancy combined with improved survival of cancer patients resulting from an increased effectiveness of cancer therapy. As the prognosis of radiation-induced malignancies in general is poor and the treatment options are limited, early diagnosis and a correspondingly better chance of complete surgical excision assume great importance. Nevertheless it has to be emphasized, that the incidence of radiation-induced malignancies is small enough that the risk of its occurrence is vastly outweighed by the benefits obtained from radiation therapy by most patients.

## References

[B1] Bluemke S (1995). Pathologie.

[B2] Adler CP (2004). Knochenkrankheiten.

[B3] Boecker W, Denk H, Heitz PU (2004). Pathologie.

[B4] Brady MS, Gaynor JJ, Brennan MF (1992). Radiation-associated sarcoma of bone and soft tissue. Arch Surg.

[B5] Patel SG, See AC, Williamson PA, Archer DJ, Evans PH (1999). Radiation induced sarcoma of the head and neck. Head Neck.

[B6] Sale KA, Wallace DI, Girod DA, Tsue TT (2004). Radiation-induced malignancy of the head and neck. Otolaryngol Head Neck Surg.

[B7] König O, Bockmühl U, Lammert I (2001). Radiation-associated malignant fibrous histiocytoma of the oropharynx. HNO.

[B8] Demirkan F, Ünal S, Cenetoglu S, Cinel L (2003). Radiation-induced leiomyosarcomas as second primary tumors in the head and neck region: report of 2 cases. J Oral Maxillofac Surg.

[B9] Dry SM, Jorgensen JL, Fletcher CDM (2000). Leiomyosarcomas of the oral cavity: an unusual topographic subset easily mistaken for nonmesenchymal tumours. Histopathology.

[B10] Oncel S, Doganay M, Ozer A, Arslanoglu S, Ermete M, Erdogan N (1996). Leiomyosarcoma of the parotid gland. J Laryngol Otol.

[B11] Frieben H (1902). Demonstration eines Cancroid des rechten Handrueckens, das sich nach langdauernder Einwirkung von Roentgenstrahlen entwickelt hatte. Fortschritte auf dem Gebiete der Roentgenstrahlen.

[B12] Cahan WG, Woodard HQ, Higinbotham NL, Steward FW, Coley BL (1948). Sarcoma arising in irradiated bone: report of eleven cases. Cancer.

[B13] Murray EM, Werner D, Greeff EA (1999). Postradiation sarcomas: 20 cases and literature review. Int J Rad Oncol Biol Phys.

[B14] Little JB (2000). Radiation carcinogenesis. Carcinogenesis.

[B15] Amendola BE, Amendola MA, McClatchey KD, Miller CH (1989). Radiation-associated sarcoma: A review of 23 patients with postradiation sarcoma over a 50 year period. Ann J Clin Oncol.

[B16] Taghian A, De Vathaire F, Terrier P, Le M, Auquier A, Mouriesse H, Grimaud E, Sarrazin D, Tubiana M (1991). Long-term risk of sarcoma following radiation treatment for breast cancer. Int J Radiat Onco Biol Phys.

[B17] Wong FL, Boice JD, Abramson DH, Tarone RE, Kleinerman RA, Stovall M, Goldman MB, Seddon JM, Tarbell N, Fraumeni JF, Li FP (1997). Cancer incidenc after retinoblastoma. Radiation dose and sarcoma risk. JAMA.

[B18] Strauss M (1983). Long-term complications of radiotherapy confronting the head and neck surgeon. Laryngoscope.

[B19] Enzinger FM, Sharon WW (1995). Soft tissue tumors.

[B20] Schenberg ME, Slootweg PT, Koole R (1993). Leiomyosarcomas of the oral cavity. Report of four cases and review of literature. J Craniomaxillofac Surg.

[B21] Sozeri B, Onerci M, Hosal S, Ruacan S (1992). Primary gingival leiomyosarcoma. J Laryngol Otol.

[B22] Rowe-Jones JM, Solomons NB, Ratcliffe NA (1994). Leiomyosarcoma of the larynx. J Laryngol Otol.

[B23] Zbaren P, Ruchti C (1994). Leiomyosarcoma of the middle ear and the temporal bone. Ann Otol Rhinol Laryngol.

[B24] van der Laan BF, Baris G, Gregor RT, Hilgers FJ, Balm AJ (1995). Radiation-induced tumours of the head and neck. J Laryngol Otol.

[B25] Miyahara H, Sato T, Yoshino K (1998). Radiation-induced cancers of the head and neck region. Acta Otolaryngol Suppl.

